# Determinants of social health in later life: the roles of family integration, social participation, and psychosocial well-being

**DOI:** 10.3389/fsoc.2026.1833784

**Published:** 2026-09-15

**Authors:** Ashifa Kariveliparambil, Fatma Sila Ayan, Alphonsa Kurian, Neethu Varghese, Maharishi Ranganathan, Jobi Babu

**Affiliations:** 1 Faculty of Health Sciences, Istanbul Gelisim University, Istanbul, Türkiye; 2 Faculty of Health Sciences, Department of Gerontology, Malatya Inonu University, Malatya, Türkiye; 3 Department of Gerontology, Institute of Health, Akdeniz University, Antalya, Türkiye; 4 Department of Sociology, Bishop Chulaparambil Memorial College Kottayam, Kottayam, India; 5 Department of English, Bishop Chulaparambil Memorial College Kottayam, Kottayam, India; 6 School of Social Sciences and Languages, Vellore Institute of Technology, Chennai, India; 7 School of Social Work, Marian College Kuttikkanam Autonomous, Idukki, Kerala, India

**Keywords:** family integration, healthy aging, older adults, psychosocial well-being, social health, social participation

## Abstract

**Introduction:**

Population aging has intensified scholarly attention towards the social dimensions of well-being in later life. While previous research has largely emphasized physical health and economic security, social health—reflecting individuals’ ability to maintain meaningful relationships and social participation—remains comparatively underexplored, particularly among older adults in rural communities. Addressing this gap, the present study investigates how family integration, social participation, and psychosocial well-being contribute to social health in later life.

**Methods:**

We conducted a quantitative, cross-sectional survey of 386 adults aged ≥60 years. Data were collected using a structured questionnaire that measured family integration, social participation, psychosocial well-being, and social health, each operationalized through multi-item Likert-type scales. Descriptive statistics, reliability analysis, Pearson’s correlation, and multiple regression analysis were employed to examine the relationships among the study variables.

**Results:**

The findings indicate that all three determinants significantly predict social health among older adults. Social participation emerged as the strongest predictor (β = 0.33, *p* < 0.001), followed by family integration (β = 0.29, *p* < 0.001) and psychosocial well-being (β = 0.27, *p* < 0.001). The regression model explained a substantial proportion of the variance in social health (R^2^ = 0.337), suggesting that relational and psychosocial resources play a critical role in shaping social well-being in later life.

**Discussion:**

The results highlight that interactions among family relationships, community engagement, and psychological resources shape social health in later life. Strengthening opportunities for social participation, reinforcing family support networks, and promoting psychosocial resilience may therefore represent key strategies for enhancing social well-being among the aging population.

## Introduction

Social health has increasingly emerged as a critical dimension of healthy aging, reflecting not only the presence of social relationships but also the capacity of individuals to remain integrated within evolving social environments across the life course (Stephens and Breheny, 2018; Holt-Lunstad, 2024; Bath and Deeg, 2005). Population aging has emerged as one of the most significant demographic transformations of the 21st century (Karaflou and Goulis, 2025). Advances in healthcare, improved living standards, and declining fertility rates have contributed to substantial increases in life expectancy across many regions of the world, resulting in a rapidly expanding older population (Bloom and Luca, 2016; Vaughan et al., 2022; Weng et al., 2025). As societies undergo this demographic transition, increasing attention has been directed toward understanding the factors that contribute to well-being and quality of life in later life. While earlier research on aging primarily emphasized physical health outcomes and economic security, contemporary scholarship increasingly recognizes that well-being in old age is multidimensional and strongly shaped by social relationships and community engagement (Bowling et al., 2003; Kim et al., 2021; Czaja et al., 2021; Monteiro et al., 2024).

From this perspective, social health has gained recognition as a critical dimension of healthy aging. Social health refers to an individual’s ability to establish and maintain meaningful interpersonal relationships, participate in social networks, and experience a sense of belonging within their community (Buja and Akhtar, 2025). For older adults, maintaining social connectedness plays an essential role in sustaining psychological stability, life satisfaction, and overall quality of life (Kuranchie, 2021; Park and Kang, 2023). Empirical studies consistently demonstrate that socially connected older individuals tend to experience lower levels of loneliness and depression, greater emotional resilience, and improved overall well-being (Hwang et al., 2020; Kamene, 2025). Conversely, social isolation has been identified as a significant risk factor for declining mental health, reduced life satisfaction, and increased mortality among the older adults (Czaja et al., 2021).

The significance of social health is particularly pronounced in rural contexts, where social environments and support structures often differ from those in urban settings. Rural communities often possess robust norms, values, and social networks that can encourage social control, social interaction, and the formation of community-level relationships (Santora et al., 2021; Cohen and Greaney, 2022). These features can create propitious surroundings in which senior citizens remain engaged with their families and communities. However, rural areas face more structural limitations than urban areas, including a lack of healthcare infrastructure, a shortage of organized social services, and geographic isolation, which may restrict opportunities for social engagement and interaction (Zhao et al., 2022; Pickering et al., 2023).

India is experiencing demographic aging due to urbanization, social transformation, and economic development. This is a modern problem that concerns research. The older adult population in India is growing, creating an increased demand for policies and social systems for healthy and active aging (Srivastava et al., 2023). Within this national context, the state of Kerala presents a unique demographic profile. The state of Kerala has one of the highest life expectancies and the largest proportion of older people in India. This indicates a significant achievement in health, education, and social development (Pengpid and Peltzer, 2021; Sebastian et al., 2021). Although these accomplishments reflect significant social progress, they also pose new challenges to social integration and well-being for older people.

Traditionally, rural communities in Kerala have been characterized by strong family systems and dense social networks that provide older adults with emotional support and opportunities for social interaction (George et al., 2021; Jose et al., 2025). However, ongoing social transformations, including the migration of younger generations to urban areas and abroad, changing family structures, and evolving economic conditions, have begun to reshape these traditional support systems (Hussain et al., 2023; Wong et al., 2025). As family structures become smaller and geographic mobility increases, older adults may experience changes in the availability of family support and opportunities for social engagement. These developments raise important questions regarding how older individuals maintain social connectedness and well-being in changing rural environments.

Previous research suggests that several factors may play a key role in shaping social well-being in later life. Family integration, reflecting the strength of relationships and interactions within family networks, provides emotional reassurance and practical assistance that support social connectedness among older adults (Cheng et al., 2022; Liu Y. et al., 2024). Social participation, referring to engagement in community activities and social organizations, enables individuals to maintain interpersonal relationships and remain actively involved in social life beyond the family sphere (Levasseur et al., 2010; Monteiro et al., 2024). In addition, psychosocial well-being, which encompasses emotional resilience and psychological stability, may influence individuals’ capacity to initiate and sustain meaningful social relationships (Kim et al., 2021; Fastame et al., 2024).

Despite the growing body of research on aging and well-being, several limitations remain in the existing literature. Much prior research has focused on physical health outcomes or economic security, despite the significant contributions of social gerontology to understanding the social dimensions of aging (Abud et al., 2022). In addition, many studies identify determinants of well-being, such as family relationships, community participation, and psychological well-being. However, they do not explore their combined influence on social health. Furthermore, a considerable proportion of research on aging has focused on the city-dwelling population, with less attention to the aging population in rural areas (Vogelsang, 2016; Weng et al., 2025; Jones et al., 2023).

This study aimed to examine the determinants of social health among older rural residents, focusing on family integration, social participation, and psychosocial well-being. Using survey data collected from 386 older adults residing in rural communities in Kerala, this study employed quantitative statistical techniques to examine the relationships among these constructs. By linking contributions from social support, active aging, and social capital theories, this study seeks a comprehensive understanding of social mechanisms for social health in later life. This study advances the literature on aging by providing new empirical evidence on the social determinants of rural older adults’ well-being. It emphasizes the interactions between relational, community, and psychological resources that sustain social health in rural areas undergoing rapid social change. Building on this conceptual understanding, the following section reviews the existing literature on social health among older adults, with particular attention to rural contexts and the theoretical foundations underpinning the study.

### Review of literature

#### Aging and social health in rural contexts

Population aging represents one of the most significant demographic transformations of the 21st century (Vogelsang, 2016; Vaughan et al., 2022; Southerland et al., 2024). Improvements in life expectancy, combined with declining fertility rates, have resulted in a rapidly expanding older population across many regions of the world (Gao et al., 2024; Weng et al., 2025). This demographic transition has prompted growing scholarly interest in understanding the determinants of well-being in later life. While earlier research primarily emphasized physical health and economic security, recent studies increasingly recognize the importance of social health as a fundamental dimension of healthy aging (Kim et al., 2021; H. Kim and Lee, 2026). Previous research has increasingly recognized social health as a central dimension of healthy aging, particularly in rural settings where family relationships, community participation, and social connectedness play an important role in maintaining well-being among older adults (Buja and Akhtar, 2025). For older adults, social health plays a critical role in sustaining psychological stability, life satisfaction, and overall quality of life (Al-Ghafri et al., 2024; J. Zhang et al., 2024). Empirical evidence consistently shows that socially connected older individuals tend to report lower levels of loneliness, improved mental health, and stronger resilience against the negative effects of aging-related transitions (Kuranchie, 2021; Miao et al., 2025). Conversely, social isolation has been identified as a major risk factor for depression, cognitive decline, and increased mortality among the older adult population (Czaja et al., 2021).

Rural contexts present distinctive opportunities and challenges for the social health of older adults. Rural communities often possess strong kinship networks, shared cultural norms, and long-standing interpersonal relationships that may foster social cohesion and collective identity (Santora et al., 2021; Cohen and Greaney, 2022; Gonçalves and Sequeira, 2024). These characteristics can provide supportive environments in which older adults remain integrated into family and community life. However, rural regions may also experience structural limitations, such as reduced access to healthcare services, limited social infrastructure, and geographic isolation (Zhao et al., 2022; Pickering et al., 2023).

Recent demographic shifts have further transformed rural social environments. The migration of younger populations toward urban areas, changing family structures, and declining population density can reduce opportunities for daily interaction and weaken traditional support systems for older adults (Cohen and Greaney, 2022; Katerenchuk and Dahlke, 2023). Consequently, maintaining social health in later life increasingly depends on the strength of family relationships, opportunities for community participation, and individuals’ psychological capacity to sustain social engagement.

Therefore, understanding these interconnected factors is essential for advancing knowledge on the social determinants of healthy aging, particularly in rural settings where social support structures are rapidly transforming (Junaedi et al., 2023; Yang et al., 2024).

#### Family integration and well-being among older adults

Family relationships remain central to social life throughout the life course. In later adulthood, family support networks are often the main sources of social and emotional support, companionship, and practical help (Thomas et al., 2017; Cheng et al., 2022; Bražinová and Chytil, 2024). Family integration among older people refers to the extent to which older people remain involved with family members through the level of contact, emotional closeness, and giving and receiving support (Silverstein and Giarrusso, 2010; Moormann et al., 2023).

Extensive research suggests that strong family integration significantly contributes to the well-being of older adults. According to Liu H. et al. (2024) and Mira et al. (2026), the support of family members leads to a sense of safety and belonging (Liu Y. et al., 2024; Mira et al., 2026). From the perspectives of Jitdorn et al. (2021) and Silverstein et al. (2025), if older people receive financial or material help that enables them to continue living independently, it is instrumental support (Jitdorn et al., 2021; Silverstein et al., 2025). Social engagement positively impacts social ties and social identity among older adults (Toyoshima and Nakahara, 2021), and family integration helps maintain social integration and mitigate isolation.

Older adults who frequently communicate with family members are emotionally stable and satisfied with their lives (Asante and Karikari, 2022; Cheng et al., 2022). Intergenerational relationships provide opportunities for shared activities, knowledge exchange, and emotional fulfillment (Liu H. et al., 2024).

Nonetheless, families are deteriorating due to social and demographic transformations in many societies. Declining household sizes, increased geographic mobility, and changing family roles have altered patterns of intergenerational interaction (Tanskanen and Danielsbacka, 2018; Fingerman et al., 2020). These developments may affect the extent to which older people stay integrated in family networks. Given the continued relevance of family relationships in later life, investigating the role of family integration in the social health of older people remains an important area of study.

#### Social participation and healthy aging

Beyond family relationships, participation in community life represents an important pathway for maintaining social well-being in older age. Social participation consists of activities that provide opportunities to interact with others in social and community settings, including involvement in local organizations, cultural events, volunteer activities, and informal social gatherings (Levasseur et al., 2010; Oe and Tadaka, 2023; Monteiro et al., 2024).

Participation in such activities allows older adults to maintain meaningful social roles and sustain interpersonal relationships beyond the immediate family. Studies suggest that social participation is positively associated with psychological well-being, life satisfaction, and cognitive functioning (Kelly et al., 2017; Holt-Lunstad, 2024; Choi et al., 2025).

Participation in community activities also contributes to the development of social networks and mutual support systems. Through these interactions, older adults exchange information, assist others, and strengthen their sense of belonging within the community (Krueger et al., 2009; Zhang et al., 2022; Long et al., 2025). Remaining active in social roles enhances identity continuity, enabling older adults to engage with and contribute positively to society.

Despite these benefits, opportunities for social participation may vary across different social and geographic contexts. Structural constraints, such as limited transportation, fewer organized programs, and physical mobility challenges, can restrict participation among older adults (Leung et al., 2021; Ong et al., 2024; Paulhus et al., 2025). Consequently, understanding the role of social participation in shaping social health remains an important focus in aging research.

#### Psychosocial well-being and social functioning

Psychosocial well-being reflects the psychological resources that enable individuals to maintain emotional balance, resilience, and adaptive functioning within social environments. For older adults, psychosocial well-being encompasses elements such as self-confidence, optimism, emotional stability, and the capacity to cope with life transitions (Fastame et al., 2024).

Research on aging consistently highlights the strong relationship between psychological well-being and social functioning. Older adults who maintain positive psychological states are more likely to engage in social interactions, develop supportive relationships, and participate in community activities (Kim et al., 2021; Au, 2025). Emotional resilience allows individuals to adapt to changes associated with aging, including retirement, declining health, and shifting family roles (Reed and Reedman, 2020).

Psychological resources also influence how individuals perceive and respond to their social environment. Older adults with higher levels of psychosocial well-being tend to exhibit greater openness to social engagement and stronger motivation to maintain interpersonal relationships (Thomas et al., 2017; Huang and Shi, 2025). Conversely, psychological distress may lead to social withdrawal and reduced participation in community life (Goll et al., 2015). Given these dynamics, psychosocial well-being represents an important internal resource that shapes individuals’ ability to sustain social connections and maintain social health in later life.

### Theoretical foundation

This study integrates social support, active aging, and social capital theories to explain the social mechanisms influencing the social health of older adults.

Social support theory posits that interpersonal relationships provide emotional, informational, and instrumental resources that enhance individuals’ capacity to cope with life challenges (Locher et al., 2004; Cherry et al., 2011; Douglas et al., 2016). For older adults, family members and social networks often function as primary support systems that reinforce social connectedness and psychological resilience (McClain et al., 2018; Guadalupe and Vicente, 2021).

Active aging theory emphasizes the importance of continued participation in social, cultural, and civic activities throughout the life course (Cornwell et al., 2008). From this perspective, aging should not be viewed as a period of social withdrawal but rather as a stage in which individuals maintain meaningful social roles through active engagement.

Social capital theory further highlights how networks of relationships generate collective resources that benefit individuals and communities. Social capital emerges through trust, reciprocity, and shared norms that facilitate cooperation among community members (Coleman, 1988; Putnam, 1993; Nyamari, 2024).

Together, these theoretical perspectives suggest that strong family relationships, active social participation, and positive psychological functioning function as complementary mechanisms shaping social health in later life.

### Research gap

Despite extensive research on aging and well-being, several gaps remain in the literature. First, much of the existing research has focused on physical health outcomes and economic security, whereas the social dimensions of aging remain comparatively underexplored (Charles et al., 2021; Abud et al., 2022; Monteiro et al., 2024). Social health, which reflects individuals’ capacity to maintain meaningful relationships and participate in social networks, has received relatively limited empirical attention.

Second, a substantial proportion of aging research has concentrated on urban populations, where institutional services and social infrastructure are more developed (Rook and Charles, 2017; Lü and Zhang, 2019; Jones et al., 2023). Rural environments present different social dynamics, characterized by strong kinship ties but fewer formal support systems. Consequently, findings derived from urban contexts may not fully capture the experiences of rural older adults.

Third, previous studies often examine the determinants of well-being in isolation. Family relationships, community participation, and psychological well-being are frequently studied as separate factors rather than as interconnected determinants of social health (Hussain et al., 2023; Yasin et al., 2025). Addressing these limitations, the present study examines the combined influence of family integration, social participation, and psychosocial well-being on the social health of rural older adults.

### Conceptual framework

Based on the theoretical perspectives outlined above, this study proposes a conceptual framework in which family integration, social participation, and psychosocial well-being serve as key determinants of social health among older adults.

Family integration represents the strength of interpersonal relationships within family networks, providing emotional support and reinforcing social belonging (Dager and McCullough, 1976; Wisnu, 2015). Social participation expands individuals’ social networks by encouraging engagement in community activities and interpersonal interactions beyond the family sphere (Levasseur et al., 2010; Kreider et al., 2015). Psychosocial well-being reflects the psychological capacity that enables individuals to initiate and sustain meaningful social relationships (Rajeev and Vijayan, 2024).

Together, these constructs represent complementary dimensions of social functioning. Strong family relationships provide foundational support (Pastor et al., 2020; Roman et al., 2025) active participation expands social engagement (Sirén et al., 2023), and psychosocial well-being enhances individuals’ capacity to maintain social connections (Fastame et al., 2024). These factors collectively contribute to the development and maintenance of social health in later life. Figure 1 presents the conceptual framework of the study.

**Figure 1:**
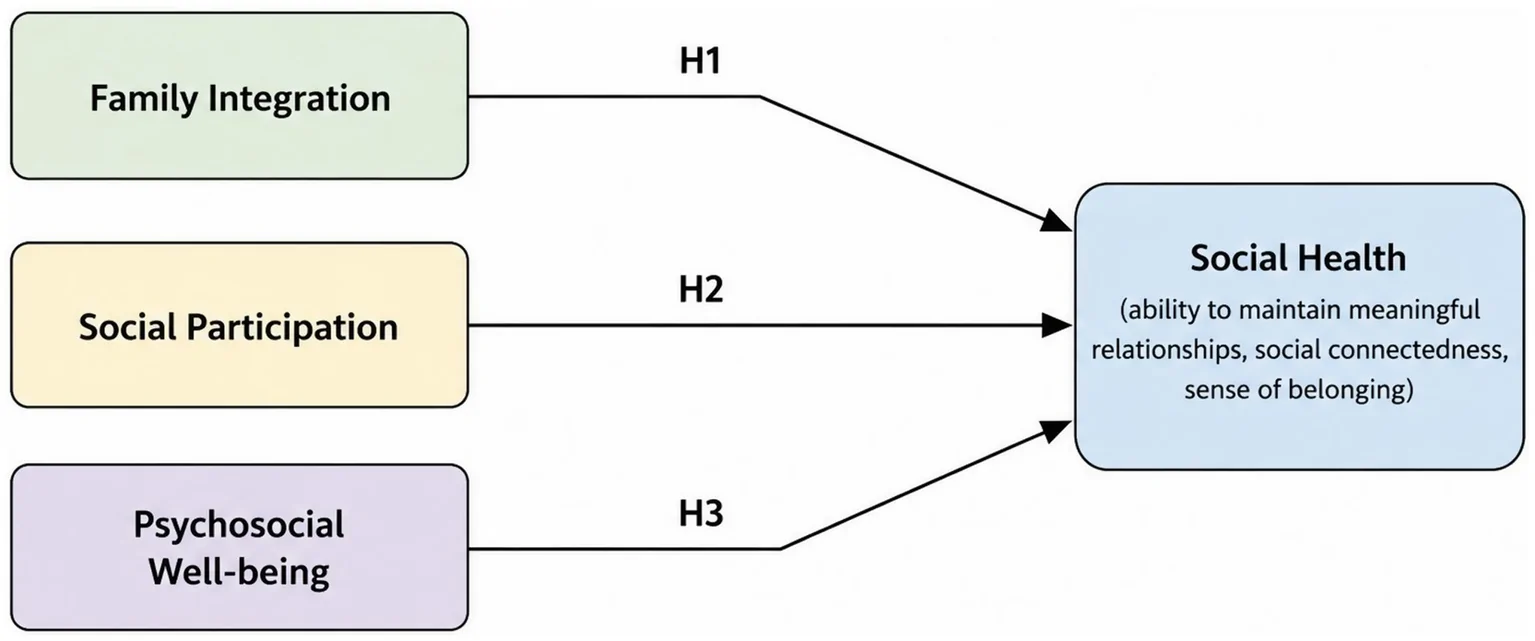
Conceptual framework of determinants of social health among rural older adults. The figure illustrates the relationships between family integration, social participation, psychosocial well-being, and social health among rural older adults.

#### Family integration and social health

Supportive family relationships play a critical role in maintaining older adults’ social well-being. Regular interaction with family members provides emotional reassurance, strengthens interpersonal bonds, and reinforces individuals’ sense of belonging (Asante and Karikari, 2022; Fernández-Portero et al., 2023). Older adults who remain actively integrated within family networks are more likely to maintain positive social identities and experience greater life satisfaction (Zheng and Chen, 2020; Cheng et al., 2022).

*H1*: Family integration positively influences social health among rural older adults.

#### Social participation and social health

Participation in community activities offers opportunities for social interaction and relationship-building. Active engagement allows older adults to expand their social networks, exchange support, and maintain meaningful roles within their communities (Levasseur and Naud, 2022; Weng et al., 2025).

*H2*: Social participation positively influences social health among rural older adults.

#### Psychosocial well-being and social health

Psychosocial well-being represents an important internal resource that influences individuals’ ability to sustain social relationships (Savchenko et al., 2023). Emotional resilience and psychological stability encourage social engagement and facilitate positive interpersonal interactions (Kim et al., 2021; Heinz et al., 2025).

*H3*: Psychosocial well-being positively influences social health among rural older adults.

## Materials and methods

### Research design

This study employed a quantitative cross-sectional research design to examine the determinants of social health among older adults residing in rural communities. A structured survey approach was used to collect primary data and analyze the relationships among family integration, social participation, psychosocial well-being, and social health. Quantitative survey designs are widely utilized in social and aging research because they allow researchers to systematically examine relationships among social and psychological variables across relatively large populations. The cross-sectional design enabled the study to capture associations among the study variables at a specific point in time and evaluate the predictive influence of key determinants of social health.

### Study setting

The study was conducted in rural villages in the Idukki district of Kerala, India, where livelihoods are primarily agricultural (Yogendra, 2024). The district is one of the most predominantly rural in the state, with dispersed settlements (Sreepriya and Balasubramanian, 2020). The district also experiences substantial migration of younger populations for education and employment, which has implications for family structures and the availability of social support for older adults (Ugargol and Bailey, 2020; Mohan et al., 2025). These demographic and social characteristics make Idukki a particularly relevant setting for examining the determinants of social health among rural older adults. The selected rural communities are characterized by close-knit family structures, strong community networks, and predominantly agrarian livelihoods, reflecting the broader socio-cultural context of rural Kerala. Although family and neighbourhood ties traditionally provide important sources of social support for older adults, demographic changes, migration of younger family members, and the gradual transition from extended to nuclear family systems have altered patterns of social interaction and community engagement. These evolving social and cultural characteristics make rural Kerala a relevant context for examining the determinants of social health among older adults, particularly in understanding how family integration, social participation, and psychosocial well-being contribute to healthy ageing.

### Sampling and data collection

Participants were selected using a community-based purposive sampling approach. The study targeted individuals aged ≥60 years who were permanent residents of rural villages in the Idukki district. Data were collected through face-to-face administration of a structured questionnaire. This approach was adopted to facilitate participation among older adults and to ensure that respondents clearly understood the survey items.

A total of 420 questionnaires were initially collected during the data collection process. Following data screening, responses that contained incomplete or missing information were excluded from the dataset. After removing the incomplete questionnaires, 386 valid responses were retained for the final statistical analysis. The final sample size was considered sufficient to conduct the planned statistical procedures, including correlation and multiple regression analyses.

### Tools for data collection

Data were collected using a structured questionnaire designed to measure the study’s key constructs. The questionnaire consisted of 16 items representing four variables: family integration, social participation, psychosocial well-being, and social health. Each construct was measured using four items. All items were measured using a five-point Likert scale ranging from 1 (strongly disagree) to 5 (strongly agree), with higher scores indicating stronger agreement with the statements and higher levels of the corresponding construct. The questionnaire was developed by the authors based on an extensive review of the literature on social relationships, family integration, social participation, psychosocial well-being, and social health among older adults, as well as the theoretical framework underpinning the study. The items were specifically developed to operationalize the study constructs in the context of rural older adults in Kerala and were not directly adapted from any single previously validated instrument.

### Measurement of variables

The study constructs were operationalized using multi-item measures designed to capture different dimensions of social interaction and well-being among older adults.

Family integration refers to the extent to which older adults remain socially and emotionally connected with their family members (Cheng et al., 2022; Silverstein and Giarrusso, 2010; Thomas et al., 2017). This construct was measured using four items assessing the frequency of interaction with family members, perceived emotional support from family members, closeness in family relationships, and a sense of belonging within the family network.

Social participation reflects older adults’ involvement in community and social activities that facilitate interactions with others (Kelly et al., 2017; Levasseur et al., 2010; Monteiro et al., 2024). This construct was measured using four items capturing participation in community gatherings, involvement in social or cultural activities, interactions with neighbors and peers, and engagement in informal social events within the community.

Psychosocial well-being was operationally defined as the individual’s capacity to maintain emotional stability, confidence in social interactions, resilience, and effective coping with everyday life challenges (Fastame et al., 2024; Kim et al., 2021). In this study, the construct was measured using four items assessing emotional stability, confidence in social interactions, ability to cope with daily challenges, and overall psychological resilience. Higher scores indicated greater psychosocial well-being.

Social health, the dependent variable in this study, refers to an individual’s ability to maintain meaningful interpersonal relationships and remain integrated within their social environment (Bath and Deeg, 2005; Buja and Akhtar, 2025; Holt-Lunstad, 2024). The construct was measured using four items capturing perceived social connectedness, satisfaction with social relationships, participation in social networks, and the ability to maintain supportive interactions with others.

### Data analysis

The collected data were analyzed using a range of statistical techniques to assess relationships among the study variables. First, descriptive statistics were computed to summarize the sociodemographic characteristics of the respondents and to examine the distribution patterns and central tendencies of the study constructs. Second, a reliability analysis was conducted to assess the internal consistency of the measurement scales using Cronbach’s alpha and McDonald’s omega coefficients. To evaluate the construct validity of the self-developed questionnaire, Exploratory Factor Analysis (EFA) was performed using Principal Axis Factoring with Varimax rotation. The suitability of the data for factor analysis was assessed using the Kaiser–Meyer–Olkin (KMO) measure of sampling adequacy and Bartlett’s Test of Sphericity. Internal consistency reliability was evaluated using Cronbach’s alpha and McDonald’s omega coefficients.

Third, Pearson’s correlation analysis was performed to examine the bivariate relationships among family integration, social participation, psychosocial well-being, and social health. Finally, multiple regression analysis was conducted to evaluate the predictive influence of the independent variables on social health, controlling for the effects of other predictors in the model.

Several diagnostic tests were conducted to ensure the robustness of the regression results. Variance inflation factor (VIF) values were examined to assess potential multicollinearity among predictors, and the Durbin–Watson statistic was used to evaluate the presence of autocorrelation among the residuals. These diagnostic procedures confirmed that the assumptions underlying the regression analysis were adequately satisfied.

### Ethical considerations

Ethical approval for the study was obtained from the Institutional Review Board (IRB) of Marian College Kuttikkanam (Autonomous), Kerala, India (MCKA/IERB/020326). The study adhered to the ethical guidelines outlined in the Declaration of Helsinki for research involving human participants. Prior to data collection, participants were informed about the purpose and procedures of the study. Informed consent was obtained from all respondents before administering the questionnaire. Participation was voluntary, and respondents were assured that their responses would remain confidential and be used solely for academic research purposes. No personally identifiable information was collected, and all data were analyzed in aggregate to ensure participant anonymity.

## Results

This section presents empirical findings from statistical analyses examining the determinants of social health among older adults. The analysis was conducted in several stages to evaluate the relationships among the study variables systematically. First, the socio-demographic characteristics of the respondents were analyzed to describe the study sample’s composition. Second, descriptive statistics were examined to understand the distribution patterns and central tendencies of the key constructs. Third, a reliability analysis was conducted to assess the internal consistency of the measurement scales. Fourth, Pearson’s correlation analysis was used to explore the bivariate relationships among the variables. Finally, multiple regression analysis was performed to determine the predictive influence of family integration, social participation, and psychosocial well-being on social health.

### Socio-demographic characteristics of respondents

Examining the socio-demographic profile of respondents provides an overview of the study sample and offers contextual information for interpreting subsequent statistical analyses. Demographic factors such as age, gender, education, income, and living arrangements are commonly reported in aging research because they can influence social relationships, access to support systems, and overall well-being.

The sociodemographic characteristics of the respondents are presented in Table 1. The study sample consisted of 386 older adults. In terms of age distribution, the largest proportion of respondents (44.6%) belonged to the 60–69 years age group, followed by 37.6% in the 70–79 years category, while 17.9% were aged 80 years and above. Regarding sex distribution, men constituted 52.6% of the sample, and women accounted for 47.4%.

**Table 1:** Socio-demographic characteristics of respondents (*N* = 386).

Variable	Category	*N*	%
Age group	60–69 years	172	44.6
	70–79 years	145	37.6
	80 years and above	69	17.9
Gender	Male	203	52.6
	Female	183	47.4
Education level	No formal education	104	26.9
	Primary education	131	33.9
	Secondary education	97	25.1
	Higher education	54	14.0
Monthly income	Low income	157	40.7
	Moderate income	146	37.8
	High income	83	21.5
Living arrangement	Living with family	248	64.2
	Living with spouse	84	21.8
	Living alone	54	14.0

*N* = 386. Values are presented as frequency (*n*) and percentage (%). Percentages represent the distribution of respondents across demographic categories.

Regarding educational attainment, 26.9% of respondents reported no formal education, 33.9% had completed primary education, 25.1% had completed secondary education, and 14.0% had completed higher education. The distribution of income levels indicates that 40.7% of participants were in the low-income group, 37.8% were in the moderate-income group, and 21.5% were in the high-income group. Regarding living arrangements, the majority of respondents (64.2%) reported living with family members, 21.8% lived with a spouse, and 14.0% lived alone. These findings suggest that a large proportion of respondents remain embedded within family-based living environments, which may influence patterns of social interaction and support among older adults.

### Descriptive statistics of study constructs

Descriptive statistics were examined to provide an overview of the central tendencies and variability of the key constructs included in the study. Understanding the distribution characteristics of the variables is an important preliminary step before conducting inferential statistical analyses, as it allows researchers to evaluate whether the data meet the assumptions required for parametric procedures.

The descriptive statistics for the study variables are presented in Table 2. The mean score for family integration was 2.98 (SD = 1.20), indicating a moderate level of perceived family connectedness among the respondents. Social participation had a mean of 3.03 (SD = 1.23), indicating that respondents reported moderate engagement in social activities within their communities. Psychosocial well-being had a mean score of 2.88 (SD = 1.19), while social health recorded a mean value of 2.90 (SD = 1.18). In addition to measures of central tendency, skewness and kurtosis were examined to assess the normality of the data distribution. The skewness values ranged from 0.05 to 0.15, while the kurtosis values ranged from −1.20 to −1.08. These values fall within the commonly accepted thresholds for normal distribution, indicating that the variables do not exhibit substantial deviation from normality. Consequently, the data are considered appropriate for subsequent parametric analyses, including correlation and regression procedures.

**Table 2:** Descriptive statistics and distribution characteristics of study constructs (*N* = 386).

Variable	M	SD	SE	Skewness	Kurtosis	Min	Max
Family integration	2.98	1.20	0.061	0.05	−1.08	1.00	5.00
Social participation	3.03	1.23	0.063	0.05	−1.20	1.00	5.00
Psychosocial well-being	2.88	1.19	0.061	0.15	−1.09	1.00	5.00
Social health	2.90	1.18	0.060	0.09	−1.13	1.00	5.00

*N* = 386. *M*, mean; SD, standard deviation; SE, standard error of the mean. Skewness and kurtosis statistics indicate that the distributions of all constructs fall within acceptable ranges for normality (skewness between −2 and +2; kurtosis between −7 and +7), supporting the use of parametric statistical analyses in subsequent stages of the study.

### Reliability and construct validity of the measurement instrument

Reliability and construct validity analyses were conducted to evaluate the psychometric properties of the self-developed questionnaire. Internal consistency was assessed using Cronbach’s alpha and McDonald’s omega coefficients, together with mean inter-item correlations and corrected item–total correlations. Construct validity was evaluated using Exploratory Factor Analysis (EFA). The results of the reliability analysis are presented in Table 3. The findings indicate that all constructs demonstrated high internal consistency. Cronbach’s alpha values ranged from 0.89 to 0.90, exceeding the commonly recommended threshold of 0.70 for acceptable reliability. Similarly, McDonald’s omega values ranged between 0.89 and 0.90, further confirming the consistency of the measurement scales. The mean inter-item correlations ranged from 0.53 to 0.57, which fall within the recommended range for assessing the homogeneity of items within a construct. Additionally, corrected item–total correlations ranged from 0.58 to 0.74 across the constructs, indicating that each item contributed meaningfully to the overall measurement of the respective variable. These results confirm that the scales measuring family integration, social participation, psychosocial well-being, and social health exhibited strong internal consistency and were suitable for further statistical analysis.

**Table 3:** Reliability and internal consistency of the measurement constructs.

Construct	Number of items	Cronbach’s α	McDonald’s ω	Mean inter-item correlation	Corrected item–total correlation (range)
Family integration	4	0.90	0.90	0.57	0.62–0.74
Social participation	4	0.90	0.90	0.55	0.60–0.72
Psychosocial well-being	4	0.89	0.89	0.53	0.58–0.71
Social health	4	0.89	0.89	0.54	0.59–0.73

Cronbach’s α and McDonald’s ω values above 0.70 indicate acceptable internal consistency. Mean inter-item correlations between 0.15 and 0.70 are considered appropriate for assessing the homogeneity of items within a construct. Corrected item–total correlations above 0.30 indicate that each indicator contributes adequately to the corresponding construct.

The results of the exploratory factor analysis are presented in Table 4. The Kaiser–Meyer–Olkin (KMO) measure indicated good sampling adequacy (KMO = 0.866), and Bartlett’s Test of Sphericity was statistically significant (χ^2^(120) = 3368.94, *p* < 0.001), confirming the suitability of the data for factor analysis. Four factors corresponding to the proposed constructs were extracted, explaining 63.70% of the total variance. Factor loadings ranged from 0.56 to 0.84, while extraction communalities ranged from 0.47 to 0.72. All items loaded strongly on their intended constructs without substantial cross-loadings, providing evidence of satisfactory construct validity for the measurement instrument.

**Table 4:** Construct validity of the measurement instrument based on exploratory factor analysis.

Construct	Item	Factor loading	Extraction communality
Family integration	FI1	0.82	0.68
	FI2	0.81	0.67
	FI3	0.84	0.71
	FI4	0.80	0.65
Social participation	SP1	0.81	0.66
	SP2	0.84	0.72
	SP3	0.79	0.64
	SP4	0.82	0.68
Psychosocial well-being	PW1	0.79	0.63
	PW2	0.81	0.66
	PW3	0.77	0.60
	PW4	0.83	0.69
Social health	SH1	0.65	0.54
	SH2	0.61	0.51
	SH3	0.58	0.49
	SH4	0.56	0.47

Construct validity was assessed using Exploratory Factor Analysis (EFA) with Principal Axis Factoring and Varimax rotation with Kaiser normalization. The Kaiser–Meyer–Olkin (KMO) measure of sampling adequacy was 0.866, indicating good sampling adequacy. Bartlett’s Test of Sphericity was statistically significant (χ^2^(120) = 3368.94, *p* < 0.001), indicating that the data were suitable for factor analysis. Four factors with eigenvalues greater than 1.0 were extracted, explaining 63.70% of the total variance. All items loaded primarily on their intended constructs, with factor loadings ranging from 0.56 to 0.84, providing evidence of satisfactory construct validity for the self-developed questionnaire.

### Pearson correlation analysis

Pearson correlation analysis was conducted to examine the bivariate relationships among the study variables. Correlation analysis provides an initial understanding of the direction and strength of associations between variables and helps identify potential multicollinearity issues before conducting regression analysis.

The correlation matrix is presented in Table 5. The results indicate that all variables are positively and significantly correlated with one another. Family integration showed a moderate positive correlation with social health (*r* = 0.43, *p* < 0.01), suggesting that stronger family connections are associated with higher levels of social health among respondents.

**Table 5:** Pearson correlation matrix of the study constructs (*N* = 386).

Variable	Family integration	Social participation	Psychosocial well-being	Social health
Family integration	1.00			
Social participation	0.29**	1.00		
Psychosocial well-being	0.34**	0.27**	1.00	
Social health	0.43**	0.43**	0.41**	1.00

*N* = 386. Values represent Pearson correlation coefficients among the study constructs. *p* < 0.01 (two-tailed). All correlations are positive and statistically significant, indicating meaningful associations among the variables examined in the study. The magnitude of the correlations remains below the commonly accepted multicollinearity threshold (*r* < 0.80), suggesting that the independent variables are sufficiently distinct while still demonstrating theoretically consistent relationships with the outcome variable.

Similarly, social participation was positively associated with social health (*r* = 0.43, *p* < 0.01), indicating that greater involvement in community activities is associated with improved social health outcomes. Psychosocial well-being was also positively correlated with social health (*r* = 0.41, *p* < 0.01), suggesting that individuals with higher psychological well-being tend to report better social health.

The correlations among the independent variables ranged from 0.27 to 0.34, indicating moderate relationships, while remaining below the commonly accepted threshold for multicollinearity concerns (*r* < 0.80). These findings suggest that the variables are conceptually related but sufficiently distinct to be included simultaneously in the regression analysis.

### Multiple regression analysis

Multiple regression analysis was performed to evaluate the predictive influence of family integration, social participation, and psychosocial well-being on social health. This analysis allowed us to examine the independent contribution of each predictor while controlling for the effects of other variables included in the model.

The regression results are presented in Table 6. The findings indicate that all three independent variables significantly predict social health. Family integration demonstrated a significant positive effect on social health (*β* = 0.29, *p* < 0.001), indicating that higher levels of family connectedness are associated with improved social health among respondents.

**Table 6:** Multiple regression analysis predicting social health (*N* = 386).

Predictor	B	SE	*β*	*t*	*p*	VIF
Constant	0.586	0.173	—	3.38	0.001	—
Family integration	0.261	0.045	0.29	5.81	<0.001	1.21
Social participation	0.279	0.043	0.33	6.55	<0.001	1.18
Psychosocial well-being	0.240	0.045	0.27	5.34	<0.001	1.24

*N* = 386. B, unstandardized regression coefficient; SE, standard error; β, standardized regression coefficient; VIF, variance inflation factor. The dependent variable in the model is social health. All predictors show statistically significant positive effects. Variance inflation factor values are below the recommended threshold of 5, indicating that multicollinearity is not a concern in the model.

Social participation emerged as the strongest predictor of social health (*β* = 0.33, *p* < 0.001), indicating that greater engagement in social and community activities is associated with higher levels of social health among older adults. Psychosocial well-being also demonstrated a significant positive effect on social health (*β* = 0.27, *p* < 0.001), suggesting that individuals with higher psychological resilience and emotional stability tend to experience improved social functioning.

The variance inflation factor (VIF) values ranged from 1.18 to 1.24, which were well below the commonly recommended threshold of 5. These results indicate that multicollinearity was not a concern in the regression model.

Model fit statistics were examined to assess the regression model’s overall explanatory power. The model fit indicators are presented in Table 7. The results indicate that the regression model explains a substantial proportion of the variance in social health.

**Table 7:** Model fit statistics for the regression model predicting social health.

Statistic	Value
R	0.581
R^2^	0.337
Adjusted R^2^	0.332
*F* (3, 382)	64.78
*p*-value	<0.001
Standard error of the estimate	0.816
Durbin–Watson	1.95
AIC	1074.00
BIC	1090.00

*N* = 386. *R*^2^ indicates the proportion of variance in social health explained by the predictors included in the model. A Durbin–Watson statistic close to 2 suggests the absence of autocorrelation in the residuals. Lower values of the Akaike Information Criterion (AIC) and Bayesian Information Criterion (BIC) indicate better model fit.

The coefficient of determination (R^2^) was 0.337, indicating that approximately 33.7% of the variance in social health is explained by the model’s predictors. The adjusted R^2^ value of 0.332 indicates that the model retains strong explanatory power even after accounting for the number of predictors.

The overall regression model was statistically significant, as indicated by the F-statistic (*F*(3, 382) = 64.78, *p* < 0.001). The Durbin–Watson statistic was 1.95, which is close to the recommended value of 2, suggesting no significant autocorrelation in the residuals. These findings indicate that the regression model demonstrates an adequate statistical fit and provides a reliable explanation of the relationships among the study variables.

### Hypothesis testing

The final stage of the analysis involved evaluating the hypotheses proposed in the conceptual framework. The hypotheses were tested using the standardized regression coefficients obtained from the multiple regression model predicting social health. The results of the hypothesis testing are summarized in Table 8.

**Table 8:** Summary of hypothesis testing results.

Hypothesis	Relationship	β	*p*-value	Decision
H1	Family Integration → Social Health	0.29	<0.001	Supported
H2	Social Participation → Social Health	0.33	<0.001	Supported
H3	Psychosocial Well-being → Social Health	0.27	<0.001	Supported

β represents standardized regression coefficients obtained from the multiple regression analysis predicting social health. All hypothesized relationships were positive and statistically significant at *p* < 0.001.

The results indicate that family integration has a significant positive effect on social health (β = 0.29, *p* < 0.001). This finding indicates that older adults with higher family connectedness are socially healthier than those with lower family connectedness. The standardized coefficient indicates a moderate positive relationship between family-based relationships and an important social resource for maintaining social functioning in later life. Therefore, H1 is supported: family integration positively impacts the social health of rural older adults.

The results also indicate that involvement in social activities has a strong positive impact on social health (*β* = 0.33, *p* < 0.001). Based on the regression model, social participation has the largest standardized coefficient, indicating that it is the strongest predictor of social health in the study. This result indicates that older people who participate in social and community employment usually rate their social health more positively. Consequently, social participation positively influences social health; therefore, H2 is supported.

Similarly, psychosocial well-being significantly predicts social health (*β* = 0.27, *p* < 0.001). The positive standardized coefficient indicates that the higher individuals’ psychological resilience and emotional stability, the better their social functioning. Although the effect size is somewhat smaller than that of the other predictors, psychosocial well-being still significantly contributes to social health in the model. Consequently, Hypothesis 3 (H3), which proposes that psychosocial well-being positively impacts social health, is supported.

The regression findings generally verify that all three hypothesized relationships are statistically significant and positively associated with social health. Psychiatric disorders are one of the crucial factors contributing to the social degradation of individuals with mental illness. Economic status is another important factor behind social degradation. These findings support the conceptual framework presented in this study.

### Subgroup analysis across demographic characteristics

To examine potential demographic heterogeneity in social health, subgroup analyses were conducted using independent-samples *t*-tests and one-way analysis of variance (ANOVA). As presented in Table 9, no statistically significant differences in social health were observed across gender (*t*(345.38) = 1.24, *p* = 0.215), age group (*F*(2, 383) = 0.50, *p* = 0.606), monthly income (*F*(2, 383) = 0.37, *p* = 0.693), or living arrangement (*F*(2, 383) = 0.53, *p* = 0.590). The corresponding effect sizes were small (Cohen’s *d* = 0.13; η^2^ = 0.002–0.003), indicating negligible differences in social health across the demographic subgroups. These findings suggest that the observed level of social health was relatively consistent across the study population, supporting the generalizability of the primary findings within the sampled rural older adults.

**Table 9:** Subgroup comparisons of social health across demographic characteristics.

Demographic variable	Group	*n*	Mean ± SD	Test statistic	*p*-value	Effect size
Gender	Male	163	3.06 ± 0.58	*t*(345.38) = 1.24	0.215	Cohen’s *d* = 0.13
	Female	223	2.99 ± 0.56			
Age group	60–69 years	187	2.99 ± 0.53	*F*(2, 383) = 0.50	0.606	η^2^ = 0.003
	70–79 years	141	3.04 ± 0.61			
	≥80 years	58	3.05 ± 0.59			
Monthly income	Below 5,000	196	3.00 ± 0.59	*F*(2, 383) = 0.37	0.693	η^2^ = 0.002
	5,000–10,000	116	3.06 ± 0.57			
	Above 10,000	74	3.00 ± 0.52			
Living arrangement	Alone	48	3.10 ± 0.56	*F*(2, 383) = 0.53	0.590	η^2^ = 0.003
	With Spouse	147	3.01 ± 0.57			
	With Children	191	3.01 ± 0.57			

Independent-samples *t*-tests were used to compare social health scores between gender groups. One-way ANOVA was used to examine differences across age groups, monthly income categories, and living arrangements. Cohen’s d is reported for the *t*-test, and eta squared (η^2^) is reported as the effect size for ANOVA.

## Discussion

This study focused on the factors affecting social health among rural older adults, including family integration, social participation, and proper psychosocial adjustment, which were found to be influential. The study findings indicate that all three predictors significantly contribute to social health. Furthermore, social participation is the strongest predictor of social health, followed by family integration and psychosocial health. These results highlight the importance of relational and community-based resources for social well-being during later life. This research, which focused on a rural population, contributes to the literature on the social determinants of healthy aging by underscoring the importance of social relationships, community engagement, and personal psychosocial resources in maintaining social health among older adults.

### Family integration and social health

The findings indicate that family integration is significant for the social health of older adults. Older adults who are socially active with family members often have better social functioning and a stronger sense of belonging. Through the transitions of aging, older people advance with the support of family members. Family bonds also provide companionship and emotional sustenance. These interactions contribute to the promotion of social bonds and the construction of social identity in later life. Previous research has consistently shown that family relationships play a vital role in the well-being of older people because of the emotional assurance exchanged between them as well as reciprocal support (Cheng et al., 2022; Liu Y. et al., 2024). The present findings support social support theory, which states that interpersonal relationships provide emotional, informational, and instrumental resources that enable individuals to cope with life’s challenges (Douglas et al., 2016; Goll et al., 2015; Oe and Tadaka, 2023). In the context of aging, family networks often function as primary support systems that reinforce social connectedness and psychological resilience. Emotional support from family members can reduce feelings of loneliness and strengthen older adults’ confidence in maintaining social relationships (Wang et al., 2020).

Earlier studies have similarly reported that older adults who maintain strong family ties often demonstrate higher levels of life satisfaction and psychological well-being (Thomas et al., 2017; Asante and Karikari, 2022). Intergenerational relationships can further enhance emotional fulfillment by facilitating knowledge exchange, shared activities, and mutual support between older and younger family members (Liu H. et al., 2024; Ugargol and Bailey, 2020). The present findings reinforce these observations by demonstrating that family integration continues to represent an important social resource for maintaining social health among older adults.

The importance of family relationships may be particularly pronounced in rural settings, where kinship networks often form the foundation of social interaction. Rural communities frequently exhibit strong interpersonal ties and shared social norms that promote intergenerational solidarity and collective responsibility (Santora et al., 2021; Cohen and Greaney, 2022). Consequently, maintaining strong family relationships can provide older adults with a stable support structure that facilitates social engagement and emotional well-being.

### Social participation and social health

*Social* participation was found to be the most significant predictor of social health in this study. The results presented show that community engagement is critical for the social functioning of older adults. Engaging in community activities helps people meet new people, visit their loved ones, and stay connected to the social world beyond the family. This finding affirms the concepts of active ageing theory, which highlights continued involvement in social, cultural, and civic activities throughout life (Cornwell et al., 2008; McClain et al., 2018; Zeng et al., 2025). In light of this ideology, aging should not be seen as a withdrawal from society but rather as a period in which older adults are functionally active. Through community engagement, seniors maintain social identities and a sense of purpose, which keeps them energized, focused, and active. Empirical evidence consistently demonstrates that participation in social and community activities is associated with improved psychological health, reduced loneliness, and enhanced life satisfaction. When citizens participate in voluntary work, social organizations, sociocultural programs, and informal gatherings, it provides them with a scope for interaction with others and a sense of belonging to the community. Social participation also contributes to the development of broader social networks and reciprocal support systems. Through regular interaction with peers and community members, older adults can exchange information, provide assistance to others, and build relationships based on trust and cooperation (Carver et al., 2018; Zeng et al., 2025). These interactions foster social cohesion and reinforce individuals’ integration within community networks (Cramm and Nieboer, 2015; Cheng et al., 2022).

The strong influence of social participation observed in this study suggests that opportunities for community engagement represent an essential component of social health in later life. In rural contexts, where institutional support services may be limited, participation in local organizations, religious groups, and community gatherings may provide crucial platforms for sustaining interpersonal relationships and maintaining social vitality among older adults (Levasseur and Naud, 2022).

### Psychosocial well-being and social health

The findings further demonstrate that psychosocial well-being significantly contributes to social health among older adults. Individuals with higher levels of psychological resilience and emotional stability are more likely to report stronger social functioning (Zábó et al., 2023; Au, 2025). Psychosocial well-being reflects internal psychological resources that enable individuals to adapt to life changes and sustain positive interpersonal relationships (Kumar, 2020; Kim et al., 2021; Fastame et al., 2024).

Previous studies have emphasized the critical role of psychological well-being in facilitating social engagement among older adults (Reed and Reedman, 2020; Au, 2025). Individuals who maintain positive emotional states often exhibit greater openness to social interaction and stronger motivation to participate in social activities. Emotional resilience enables individuals to cope with life transitions, such as retirement, health challenges, or changes in family roles, while maintaining social engagement.

Psychological resources also influence how individuals perceive and respond to their social environment (Thomas et al., 2017; Portegijs et al., 2023). Older adults with higher psychosocial well-being are more likely to initiate and maintain interpersonal relationships, participate in community activities, and remain socially integrated within their networks (Huang and Shi, 2025). Conversely, psychological distress may lead to social withdrawal and reduced participation in social life (Saïas et al., 2014).

Therefore, the results of this study suggest that psychosocial well-being functions as an important internal resource that supports the development and maintenance of social relationships in later life. By fostering emotional stability and resilience, psychological well-being enhances individuals’ ability to sustain social connections and actively participate in community life.

Taken together, these findings suggest that social health among older adults is shaped by the interaction of relational, community, and psychological resources. Family integration provides foundational emotional support, social participation expands interpersonal networks, and psychosocial well-being strengthens individuals’ capacity to sustain meaningful social relationships (An et al., 2024; Zhang and Sun, 2024). These factors operate as complementary mechanisms that collectively influence the social well-being of older adults.

This integrated perspective closely aligns with social capital theory, which emphasizes how networks of relationships generate collective resources that benefit individuals and communities (Coleman, 1988; Putnam, 1993; Nyamari, 2024). Through active engagement in family and community networks, older adults accumulate social capital that enhances their access to support, strengthens social trust, and reinforces a sense of belonging.

Additional subgroup analyses indicated that social health did not differ significantly across gender, age group, monthly income, or living arrangement. These findings suggest that the associations between family integration, social participation, psychosocial well-being, and social health were broadly consistent across the demographic subgroups included in this study. Nevertheless, future research involving more diverse populations and larger samples may further explore potential demographic heterogeneity and contextual differences in the determinants of social health among older adults.

Therefore, the findings suggest that social health in later life cannot be understood solely in terms of individual characteristics. Rather, it emerges through the interaction between personal psychological resources and broader social environments. Strong family relationships, active participation in community life, and positive psychological functioning collectively contribute to the maintenance of social well-being among older adults. Although the regression model explained 33.7% of the variance in social health, a substantial proportion of the variance remained unexplained, indicating that social health is influenced by additional factors beyond family integration, social participation, and psychosocial well-being. Variables such as physical health status, functional ability, economic security, neighbourhood environment, access to healthcare and social services, digital inclusion, and broader community resources may also contribute to social health in later life. Future research should incorporate these contextual and structural determinants to develop a more comprehensive understanding of healthy ageing among rural older adults.

### Theoretical contributions

This study contributes to the growing body of research on aging and well-being by advancing the conceptual understanding of social health among older adults. While a substantial proportion of aging research has traditionally emphasized physical health, healthcare access, and economic security, comparatively less attention has been devoted to the social dimensions of well-being in later life (Abud et al., 2022; Monteiro et al., 2024). By examining social health as a multidimensional construct shaped by interpersonal relationships, community engagement, and psychological resources, the present study expands the theoretical discourse on the social determinants of healthy aging.

First, the findings extend the application of social support theory by demonstrating the continuing importance of family integration as a foundational component of social well-being among older adults. Social support theory suggests that interpersonal relationships provide emotional, informational, and instrumental resources that enhance individuals’ ability to cope with life challenges (Cherry et al., 2011; Douglas et al., 2016). The results of this study reinforce this perspective by showing that older adults who maintain stronger connections with family members experience better social health. These findings highlight the role of family networks as essential support systems that strengthen emotional security, social connectedness, and interpersonal engagement in later life (Cheng et al., 2022; Liu Y. et al., 2024).

Second, this study contributes empirical evidence to active aging theory, which emphasizes the importance of sustained participation in social, cultural, and civic activities throughout the life course (Cornwell et al., 2008; McClain et al., 2018). The finding that social participation was the strongest predictor of social health underscores the importance of maintaining active social roles as individuals age. This result reinforces the theoretical argument that successful aging involves continued engagement in meaningful social activities rather than withdrawal from social life. Participation in community networks allows older adults to maintain social identity, expand interpersonal relationships, and sustain a sense of purpose (Levasseur et al., 2010; Kelly et al., 2017; Holt-Lunstad, 2024).

Third, this study advances the application of social capital theory in the context of aging by illustrating how networks of relationships generate social resources that contribute to well-being. Social capital emerges through patterns of trust, reciprocity, and shared norms that facilitate cooperation within communities (Coleman, 1988; Putnam, 1993; Nyamari, 2024; Svendsen and Svendsen, 2000). The findings suggest that family relationships and community participation function as key mechanisms through which older adults accumulate social capital, thereby strengthening their capacity to maintain supportive social networks and sustain social health.

By integrating social support theory, active aging theory, and social capital theory, the present study provides a comprehensive framework for understanding the social mechanisms that influence well-being in later life. Rather than examining family relationships, social participation, and psychological resources as independent determinants, the findings demonstrate how these dimensions operate interactively to shape social health among older adults. This integrated perspective contributes to the broader literature on aging by illustrating that social health emerges through the dynamic interaction between personal resources and social environments.

### Policy and practical implications

The findings of this study have important implications for policymakers, practitioners, and community organizations working to promote healthy aging. A rapidly aging global population has made the social well-being of older people a growing public health priority (Khan et al., 2024; Au, 2025). First, the results highlight the importance of strengthening family-based support systems for older adults. Policies can enhance social well-being and bolster emotional support networks while limiting the risk of social isolation (Zlotnick et al., 2020; Zeng et al., 2025) by promoting intergenerational interaction and family involvement in elder care. Community programs that engage family members can strengthen relationships and improve the quality of life of older adults. Direct support and counselling programs offer caregivers help in the course of their caregiving tasks.

Further, engaging older people in the community with children and families is a great way to reduce loneliness. Second, the strong influence of social participation suggests that community engagement opportunities play a critical role in sustaining social health among older adults. To encourage older people to participate in social organizations, volunteer, cultural, and neighborhood associations, governments and local organizations may wish to develop age-friendly environments (Portegijs et al., 2023). These initiatives help strengthen the connections among the older adults with their community and other people.

Third, the findings underscore the importance of promoting psychosocial well-being as a key component of healthy aging. Mental health programs, resilience-building interventions, and counselling services can help older adults maintain emotional stability and adapt to life transitions associated with aging (Zábó et al., 2023; Kamene, 2025). Supporting psychological well-being may indirectly enhance social health by encouraging older adults to remain socially engaged and actively participate in community life.

In conclusion, these implications suggest that promoting social health among older adults requires a holistic approach that integrates family support, community participation, and psychological well-being. Policies that strengthen these interconnected dimensions may significantly contribute to improving the quality of life and social integration of the aging population.

### Limitations and future research directions

Despite its contributions, the present study has several limitations that should be acknowledged. First, the study employed a cross-sectional research design, which limits the ability to establish causal relationships between the variables. Although the results indicate significant associations between family integration, social participation, psychosocial well-being, and social health, longitudinal studies would be valuable for examining how these relationships evolve. Although the conceptual framework was informed by Social Support Theory, Active Aging Theory, and Social Capital Theory, which suggest potentially complex relationships among family integration, social participation, psychosocial well-being, and social health, the present study examined only the direct additive effects of these variables using multiple regression analysis. Consequently, potential mediating or moderating mechanisms were not investigated. Future research should employ more advanced analytical approaches, such as mediation analysis, moderation analysis, or structural equation modelling, to examine the indirect and interactive pathways through which these factors influence social health among older adults.

Second, the research used survey data on self-report, which may have had biases owing to the respondents’ assertions. The ways in which participants evaluated their social relationships or psychological well-being may have led to socially desirable responses. In future studies, the addition of observational measures or qualitative interviews may offer a richer understanding of social health among older adults through enhanced data sources.

The third limitation is that the data were collected in a specific rural context, raising questions about the applicability of the findings to other geographic or cultural contexts. Social relationships and community structures may differ across locations, highlighting the importance of understanding contrasting relationships in other rural and urban environments in future research. Although this study identified three determinants of social health, other structural and environmental determinants may enhance social well-being in later years. Older adults social engagement may depend on their physical health status, access to technology, neighborhood, and availability of social services.

Future studies may include additional contextual variables to better understand social health determinants. Finally, future studies can benefit from the use of mixed methodologies, including both quantitative and qualitative methods. Qualitative investigations could provide a deeper understanding of the experiences of older adults and the impact of social relationships and community engagement on everyday well-being. Despite these limitations, the current study contributes to the literature on the social determinants of well-being among rural older adults. It also emphasizes the importance of strengthening family bonds, community engagement, and psychological resources to promote healthy aging.

## Conclusion

The present investigation analyzed the determinants of social health of rural older adults. And it examined family integration, social participation and, psychosocial well-being. It was found that all three variables significantly influenced social health, with social participation being the most significant predictor, followed by family integration and psychosocial well-being. This finding highlights the significance of both social relationships and community involvement in achieving social well-being in old age. This study contributes to the literature on aging by emphasizing that social health is shaped through the interaction of relational, community, and psychological resources. Having strong family relationships provides emotional support and opportunities for community activity, which helps develop social networks and generate a sense of belonging. Similarly, psychosocial well-being builds individuals’ capacity to have meaningful relationships and remain socially engaged. Drawing on social support, active aging, and social capital theories, this study offers a well-rounded perspective on the social mechanisms that drive well-being among older adults. Promoting family connectedness, participation in communities, and strengthening psychological resilience are important strategies for healthy and socially engaged aging, according to the findings. Overall, the study’s findings suggest that the social health and quality of life of older adults in rural communities can improve significantly with a supportive social environment and active community engagement.

## Data Availability

The raw data supporting the conclusions of this article will be made available by the authors, without undue reservation.

## References

[ref1] Abud T. Kounidas G. Martin K. R. Werth M. Cooper K. Myint P. K. (2022). Determinants of healthy ageing: a systematic review of contemporary literature. Aging Clin. Exp. Res. 34, 1215–1223. doi: 10.1007/s40520-021-02049-w PMC8821855 35132578

[ref2] Al-Ghafri B. R. Sinawi H. A. Al-Harrasi A. Alsaidi Y. Al-Mahrezi A. Ghusaini Z. B. A. . (2024). Effects of life-story review on quality of life, depression, and life satisfaction in older adults in Oman: a randomized controlled study. BMC Geriatr. 24:530. doi: 10.1186/s12877-024-05133-8 PMC11188283 38898425

[ref3] An J. Zhu X. Shi Z. An J. (2024). A serial mediating effect of perceived family support on psychological well-being. BMC Public Health 24:38566105. doi: 10.1186/s12889-024-18476-z, 38566105 PMC10986067

[ref4] Asante S. Karikari G. (2022). Social relationships and the health of older adults: an examination of social connectedness and perceived social support. J. Ageing Longev. 2, 49–62. doi: 10.3390/jal2010005

[ref5] Au D. W. H. (2025). Psychological health and social wellbeing among older adults. Healthcare 14, 65–65. doi: 10.3390/healthcare14010065 PMC12785500 41516996

[ref6] Bath P. A. Deeg D. (2005). Social engagement and health outcomes among older people: introduction to a special section. Eur. J. Ageing 2, 24–30. doi: 10.1007/s10433-005-0019-4, 28794713 PMC5547666

[ref7] Bloom D. E. Luca D. L. (2016). The global demography of aging: facts, explanations, future. RePEc: Research papers in economics. Available online at: https://econpapers.repec.org/RePEc:iza:izadps:dp10163 (Accessed February 23, 2026).

[ref8] Bowling A. Gabriel Z. Dykes J. Dowding L. M. Evans O. Fleissig A. . (2003). Let’s ask them: a national survey of definitions of quality of life and its enhancement among people aged 65 and over. Int. J. Aging Hum. Dev. 56, 269–306. doi: 10.2190/bf8g-5j8l-ytrf-6404 14738211

[ref9] Bražinová I. Chytil O. (2024). The family as a source of social support for older adults: implications for gerontological social work. J. Soc. Work. 24, 339–356. doi: 10.1177/14680173231222612

[ref10] Buja A. Akhtar S. (2025). The association between social capital and quality of life in old adults: a systematic review and meta-analysis. Front. Public Health 13:1668696. doi: 10.3389/fpubh.2025.1668696 PMC12629930 41283054

[ref11] Carver L. Beamish R. Phillips S. P. Villeneuve M. (2018). A scoping review: social participation as a cornerstone of successful aging in place among rural older adults. Geriatrics 3, 75–75. (31011110). doi: 10.3390/geriatrics3040075, 31011110 PMC6371105

[ref12] Charles S. T. Röcke C. Zadeh R. S. Martin M. Boker S. Scholz U. (2021). Leveraging daily social experiences to motivate healthy aging. J Gerontol B Psychol Sci Soc Sci 76, S157–S166. doi: 10.1093/geronb/gbab028, 33861858

[ref13] Cheng W. Song W. Ye C. Wang Z. (2022). Family networks, social networks, and life satisfaction of older adults in China. Healthcare 10, 1568–1568. (36011225). doi: 10.3390/healthcare10081568, 36011225 PMC9407943

[ref14] Cherry K. E. Walker E. J. Brown J. S. Vólaufová J. LaMotte L. R. Welsh D. A. . (2011). Social engagement and health in younger, older, and oldest-old adults in the Louisiana healthy aging study. J. Appl. Gerontol. 32, 51–75. (23526628). doi: 10.1177/0733464811409034, 23526628 PMC3604902

[ref15] Choi N. G. Marti C. N. Zhou Y. Kunik M. E. (2025). Social participation and psychological well-being among older adults with dementia. Dementia 1–23. doi: 10.1177/14713012251375273 PMC13569912 40882214

[ref16] Cohen S. A. Greaney M. L. (2022). Aging in rural communities. Curr. Epidemiol. Rep. 10, 1–16. (36404874). doi: 10.1007/s40471-022-00313-9, 36404874 PMC9644394

[ref17] Coleman J. S. (1988). Social capital in the creation of human capital. Am. J. Sociol. 94, S95–S120. doi: 10.1086/228943

[ref18] Cornwell B. Laumann E. O. Schumm L. P. (2008). The social connectedness of older adults: a National Profile. Am. Sociol. Rev. 73, 185–203. (19018292). doi: 10.1177/000312240807300201, 19018292 PMC2583428

[ref19] Cramm J. M. Nieboer A. P. (2015). Social cohesion and belonging predict the well-being of community-dwelling older people. BMC Geriatr. 15:30. doi: 10.1186/s12877-015-0027-y, 25879773 PMC4369354

[ref20] Czaja S. J. Moxley J. Rogers W. (2021). Social support, isolation, loneliness, and health among older adults in the PRISM randomized controlled trial. Front. Psychol. 12, 728658–728658. (34675843). doi: 10.3389/fpsyg.2021.728658, 34675843 PMC8525598

[ref21] Dager E. Z. McCullough B. C. (1976). Family Integration: A Multi-Dimensional Concept. College Park, MD: University of Maryland, Computer Science Center.

[ref22] Douglas H. Georgiou A. Westbrook J. (2016). Social participation as an indicator of successful aging: an overview of concepts and their associations with health. Aust. Health Rev. 41, 455–455. doi: 10.1071/ah16038 27712611

[ref23] Fastame M. C. Manca C. Mulas I. Ruiu M. (2024). Psychosocial correlates of flourishing in the late lifespan. Aging Clin. Exp. Res. 36:68. doi: 10.1007/s40520-024-02703-z, 38480617 PMC10937776

[ref24] Fernández-Portero C. Amián J. G. Alarcón D. Villalba M. J. A. Sánchez-Medina J. A. (2023). The effect of social relationships on the well-being and happiness of older adults living alone or with relatives. Healthcare 11, 222–222. (36673590). doi: 10.3390/healthcare11020222, 36673590 PMC9858722

[ref25] Fingerman K. L. Huo M. Birditt K. S. (2020). A decade of research on intergenerational ties: technological, economic, political, and demographic changes. J. Marriage Fam. 82, 383–403. (38831801). doi: 10.1111/jomf.12604, 38831801 PMC11145410

[ref26] Gao M. Jiang F. Wang J. Wu B. (2024). Population ageing and income inequality in rural China: an 18-year analysis. Humanit. Soc. Sci. Commun. 11:1605. doi: 10.1057/s41599-024-04110-1

[ref27] George M. S. Gaitonde R. Davey R. Sukumaran V. Mohanty I. Upton P. (2021). Social networks and their impact on access to health care: insights from older widows living alone in Kottayam, South India. Ageing Soc. 43, 1141–1163. doi: 10.1017/s0144686x21001100

[ref28] Goll J. C. Charlesworth G. Scior K. Stott J. (2015). Barriers to social participation among lonely older adults: the influence of social fears and identity. PLoS One 10:30044856. doi: 10.1371/journal.pone.0116664, 25706933 PMC4338142

[ref29] Gonçalves H. Sequeira T. (2024). Quality of life and aging in place in rural areas: what community social support systems? Rev. Gestão Soc. Ambient. 18:e09800. doi: 10.24857/rgsa.v18n11-132

[ref30] Guadalupe S. Vicente H. T. (2021). “Intergenerational support networks and wellbeing in old age,” in IntechOpen eBooks, Editor. Yonk RM, (London, United Kingdom: IntechOpen).

[ref31] Heinz S. S. O’Brien A. Walker C. O’Sullivan M. Rouse P. Whitehead J. . (2025). Mediating pathways between resilience, mental health and wellbeing: a scoping review of individual, social, and systemic factors. BMC Public Health 25, 3758–3758. doi: 10.1186/s12889-025-24897-1, 41184791 PMC12581430

[ref32] Holt-Lunstad J. (2024). Social connection as a critical factor for mental and physical health: evidence, trends, challenges, and future implications. World Psychiatry 23, 312–332. (39279411). doi: 10.1002/wps.21224, 39279411 PMC11403199

[ref33] Huang J. Shi Z. (2025). Exploring the interaction between self-rated health, intergenerational relationship, and subjective well-being in older adults: an empirical analysis. INQUIRY J. Health Care Organ. Provis. Financ. 62:469580251399393. doi: 10.1177/00469580251399393 PMC12665019 41316944

[ref34] Hussain B. Mirza M. Baines R. Burns L. Stevens S. Asthana S. . (2023). Loneliness and social networks of older adults in rural communities: a narrative synthesis systematic review. Front. Public Health 11:1113864. doi: 10.3389/fpubh.2023.1113864 PMC10225733 37255758

[ref35] Hwang J. Toma C. L. Chen J. Shah D. V. Gustafson D. H. Mares M. (2020). Effects of web-based social connectedness on older adults’ depressive symptoms: a two-wave cross-lagged panel study. J. Med. Internet Res. 23:e21275. doi: 10.2196/21275 PMC7840281 33439143

[ref36] Jitdorn J. Somprasert C. Kongvattananon P. Soonthornchaiya R. (2021). An integrative review of experience of well-being and happiness: insights from older adults in the community. Trends Sci. 18, 20–20. doi: 10.48048/tis.2021.20

[ref37] Jones C. A. Jhangri G. S. Yamamoto S. Hogan D. B. Hanson H. Levasseur M. . (2023). Social participation of older people in urban and rural areas: Canadian longitudinal study on aging. BMC Geriatr. 23, 439–439. (37464306). doi: 10.1186/s12877-023-04127-2, 37464306 PMC10354920

[ref38] Jose P. Basheer M. T. B. Prasad D. S. Varma R. P. (2025). Do current “age-friendly” interventions enable civic participation of community dwelling older adults? A mixed methods study from rural Kerala. J. Soc. Econ. Dev. 28, 160–179. doi: 10.1007/s40847-025-00475-4

[ref39] Junaedi J. Dikrurohman D. Abdullah A. (2023). Analysis of social change in rural communities due to the influence of urbanization and globalization in Indonesia. Edunity Kajian Ilmu Sosial Dan Pendidikan 2, 431–441. doi: 10.57096/edunity.v2i3.76

[ref40] Kamene K. (2025). Graceful aging: addressing cognitive and emotional challenges in later life. Premier J. Psychol. 4:100009 doi: 10.70389/pjp.100009

[ref41] Karaflou M. Goulis D. G. (2025). The growing challenge of aging populations: addressing health and societal impacts. Maturitas 202, 108720–108720 (40934636). doi: 10.1016/j.maturitas.2025.108720, 40934636

[ref42] Katerenchuk J. Dahlke S. (2023). Strength and vulnerability of mental illness in older persons within the rural context. Online J. Rural Nurs. Health Care 23, 70–130. doi: 10.14574/ojrnhc.v23i1.725

[ref43] Kelly M. E. Duff H. Kelly S. Power J. M. Brennan S. Lawlor B. . (2017). The impact of social activities, social networks, social support and social relationships on the cognitive functioning of healthy older adults: a systematic review. Syst. Rev. 6, 259–259. (29258596). doi: 10.1186/s13643-017-0632-2, 29258596 PMC5735742

[ref44] Khan H. T. A. Addo K. M. Findlay H. (2024). Public health challenges and responses to the growing ageing populations. Public Health Challenges 3:e213. doi: 10.1002/puh2.213, 40496520 PMC12039680

[ref45] Kim H. Lee B. (2026). Exploring the determinants of subjective well-being in older adults: a cross-national analysis of social, political, economic, and demographic factors. J. Happiness Stud. 27:30. doi: 10.1007/s10902-026-01010-w

[ref46] Kim E. S. Tkatch R. Martin D. MacLeod S. Sandy L. G. Yeh C. S. (2021). Resilient aging: psychological well-being and social well-being as targets for the promotion of healthy aging. Gerontol. Geriatr. Med. 7:23337214211002951. doi: 10.1177/23337214211002951, 33816707 PMC7995285

[ref47] Kreider C. Bendixen R. Young M. E. Prudencio S. M. McCarty C. Mann W. C. (2015). Social networks and participation with others for youth with learning, attention, and autism spectrum disorders. Can. J. Occup. Ther. 83, 14–26. (26755040). doi: 10.1177/0008417415583107, 26755040 PMC4710853

[ref48] Krueger K. R. Wilson R. S. Kamenetsky J. M. Barnes L. L. Bienias J. L. Bennett D. A. (2009). Social engagement and cognitive function in old age. Exp. Aging Res. 35, 45–60. (19173101). doi: 10.1080/03610730802545028, 19173101 PMC2758920

[ref49] Kumar C. (2020). “Psychosocial well-being of individuals,” in Encyclopedia of the UN Sustainable Development Goals, eds. Filho W. L. Azul A. M. Brandli L. Özuyar P. G. Wall T. (Cham: Springer International Publishing), 676–686.

[ref50] Kuranchie A. (2021). Social connectedness of the older adults in non-urban settings: an empirical evidence. J. Soc. Sci. Humanit. 60, 39–55. doi: 10.46568/jssh.v60i2.548

[ref51] Leung A. Y. M. Molassiotis A. Carino D. A. (2021). A challenge to healthy aging: limited social participation in old age. Aging Dis. 12, 1536–1538. doi: 10.14336/ad.2021.02018, 34631202 PMC8460313

[ref52] Levasseur M. Naud D. (2022). “The Application of Salutogenesis for Social Support and Participation: Toward Fostering Active and Engaged Aging at Home,” in The Handbook of Salutogenesis. Eds. M. S. Mittelmark, G. F. Bauer, L. Vaandrager, J. M. Pelikan, S. Sagy, M. Eriksson, et al., (Cham: Springer), 249–258. 36122029

[ref53] Levasseur M. Richard L. Gauvin L. Raymond É. (2010). Inventory and analysis of definitions of social participation found in the aging literature: proposed taxonomy of social activities. Soc. Sci. Med. 71, 2141–2149. (21044812). doi: 10.1016/j.socscimed.2010.09.041, 21044812 PMC3597625

[ref54] Liu Y. Me R. C. Yus I. S. M. (2024). Emotional needs for smart products: a case study of older people living alone in Chengdu, China. Research Square (Research Square). doi: 10.21203/rs.3.rs-5332088/v1 PMC12146300 40491943

[ref55] Liu H. Topping A. Guo P. (2024). Intergenerational engagement with Asian residents in long-term care facilities: a mixed method systematic review. Front. Public Health 12:1422134. doi: 10.3389/fpubh.2024.1422134 PMC11286590 39081361

[ref56] Locher J. L. Ritchie C. S. Roth D. L. Baker P. S. Bodner E. Allman R. M. (2004). Social isolation, support, and capital and nutritional risk in an older sample: ethnic and gender differences. Soc. Sci. Med. 60, 747–761. (15571893). doi: 10.1016/j.socscimed.2004.06.023, 15571893 PMC2763304

[ref57] Long Y. Li X. Ben-xian Y. Liu Y. Xu H. (2025). The relationship between community spiritual comfort services and cognitive function in the older adults: a random intercept cross-lagged panel study. Eur. J. Ageing 22:52. doi: 10.1007/s10433-025-00885-7 PMC12554857 41139715

[ref58] Lü N. Zhang J. (2019). Social capital and self-rated health among older adults living in urban China: a mediation model. Sustainability 11, 5566–5566. doi: 10.3390/su11205566

[ref59] McClain J. Gullatt K. Lee C. H. (2018) Resilience and Protective Factors in Older Adults. San Rafael, CA; Dominican University of California.

[ref60] Miao M.-Y. Fang F. Lyu J. Liu Z.-Y. Wan Z. Qin L.-Q. . (2025). Relationships of social isolation and loneliness with healthy aging among older adults. BMC Geriatr. 25, 267–267. doi: 10.1186/s12877-025-05941-6, 40269703 PMC12016190

[ref61] Mira P. Turja T. Mari A. Johanna E. Marja K. (2026). The sense of security and associated factors among older home care clients: a register study. Scand. J. Caring Sci. 40:e70187. doi: 10.1111/scs.70187 PMC12808853 41540657

[ref62] Mohan A. Jothikaran T. A. J. Patil D. S. Ashok L. (2025). ‘Care beyond co-residence’: a qualitative exploration of emotional and instrumental care gaps among older adults in migrant households of Kerala. Int. J. Environ. Res. Public Health 22, 1745–1745. (41302691). doi: 10.3390/ijerph22111745, 41302691 PMC12652767

[ref63] Monteiro J. Gonçalves R. Bastos A. Barbosa R. (2024). Social engagement and wellbeing in late life: a systematic review. Ageing Soc. 45, 1327–1354. doi: 10.1017/s0144686x24000011

[ref64] Moormann K. I. Pabst A. Bleck F. Löbner M. Kaduszkiewicz H. van der Leeden C. . (2023). Social isolation in the oldest-old: determinants and the differential role of family and friends. Soc. Psychiatry Psychiatr. Epidemiol. 59, 979–988. (37407737). doi: 10.1007/s00127-023-02524-x, 37407737 PMC11116196

[ref65] Nyamari T. (2024). Social capital and community development. Int. J. Humanit. Soc. Sci. 3, 14–27. doi: 10.47941/ijhss.1890

[ref66] Oe N. Tadaka E. (2023). Development of the self-efficacy for social participation scale (SOSA) for community-dwelling older adults. BMC Public Health 23:2294. doi: 10.1186/s12889-023-16774-6, 37985978 PMC10662651

[ref67] Ong C. H. Pham B. L. Levasseur M. Tan G. R. Seah B. (2024). Sex and gender differences in social participation among community-dwelling older adults: a systematic review. Front. Public Health 12:1335692. doi: 10.3389/fpubh.2024.1335692 PMC11046488 38680931

[ref68] Park J. H. Kang S. (2023). Social interaction and life satisfaction among older adults by age group. Healthcare 11, 2951–2951. doi: 10.3390/healthcare11222951, 37998443 PMC10671417

[ref69] Pastor D. H. Frost N. Devaney C. (2020). Understanding contemporary family support: reflections on theoretical and conceptual frameworks. Soc. Work. Soc. Sci. Rev. 21, 27–45. doi: 10.1921/swssr.v21i2.1420

[ref70] Paulhus L. Bernard K. Bourque C. J. Guilmette S. Laquerre M.-Ê. Laramée e. L.-È. . (2025). An updated synthesis of environmental factors influencing mobility and social participation in older adults: results from a scoping review. BMC Public Health 26, 109–109. doi: 10.1186/s12889-025-25849-5 PMC12781921 41345601

[ref71] Pengpid S. Peltzer K. (2021). Successful ageing among a national community-dwelling sample of older adults in India in 2017–2018. Sci. Rep. 11:22186. doi: 10.1038/s41598-021-00739-z, 34772955 PMC8589844

[ref72] Pickering J. Wister A. O’Dea E. Chaudhury H. (2023). Social isolation and loneliness among older adults living in rural areas during the COVID-19 pandemic: a scoping review. BMC Geriatr. 23:511. doi: 10.1186/s12877-023-04196-3 PMC10464360 37612647

[ref73] Portegijs E. Lee C. Zhu X. (2023). Activity-friendly environments for active aging: the physical, social, and technology environments. Front. Public Health 10:1080148. doi: 10.3389/fpubh.2022.1080148, 36711401 PMC9875594

[ref74] Putnam R. D. (1993). The prosperous community: social capital and public life. The American Prospect 13, 65–78.

[ref75] Rajeev N. Vijayan D. (2024). Social connectedness and psychological well-being among divorced women. Int. J. Res. Appl. Sci. Eng. Technol. 12, 3178–3184. doi: 10.22214/ijraset.2024.60577

[ref76] Reed D. E. Reedman A. E. (2020). Reactivity and adaptability: applying gender and age assessment to the leader resilience profile®. Front. Educ. 5:574079. doi: 10.3389/feduc.2020.574079

[ref77] Roman N. V. Balogun T. V. Butler-Kruger L. Danga S. D. Lange J. T. Human-Hendricks A. . (2025). Strengthening family bonds: a systematic review of factors and interventions that enhance family cohesion. Soc. Sci. 14, 371–371. doi: 10.3390/socsci14060371

[ref78] Rook K. S. Charles S. T. (2017). Close social ties and health in later life: strengths and vulnerabilities. Am. Psychol. 72, 567–577. doi: 10.1037/amp0000104, 28880103 PMC5599123

[ref79] Saïas T. Roscoät E. Véron L. Guignard R. Richard J. Legleye S. . (2014). Psychological distress in French college students: demographic, economic and social stressors. Results from the 2010 National Health Barometer. BMC Public Health 14:256. doi: 10.1186/1471-2458-14-256 PMC3995499 24629002

[ref80] Santora L. Byrne D. Klöckner C. A. (2021). Exploring quality of life reported by Norwegian older adults using classification tree approach on group profiles. Ageing Int. 47, 816–846. doi: 10.1007/s12126-021-09454-6

[ref81] Savchenko O. V. Korvat L. Lavrynenko D. (2023). “Resourcefulness as an important Indicator of personal well-being,” in IntechOpen eBooks. Eds. J. Mullings, T. Paul, L. Dunn, S. Arbor, J. Meeks-Gardner, and T. Arbor, (London, United Kingdom: IntechOpen).

[ref82] Sebastian I. R. Venkitaraman S. Syamala S. Shajan A. Tennyson N. Devi Y. (2021). Nutritional status and COVID-19 among older persons in Kerala, India. Inquiry 58:00469580211059738. doi: 10.1177/00469580211059738, 34870520 PMC8842181

[ref83] Silverstein M. Fingerman K. L. Suitor J. J. (2025). Intergenerational relationships and family support: evidence for health and well-being in studies supported by the National Institute on Aging. The Gerontologist 65:gnaf003. doi: 10.1093/geront/gnaf003, 39780401

[ref84] Silverstein M. Giarrusso R. (2010). Aging and family life: a decade review. J. Marriage Fam. 72, 1039–1058. doi: 10.1111/j.1741-3737.2010.00749.x, 22930600 PMC3427733

[ref85] Sirén A. L. Seppänen M. von Bonsdorff M. B. (2023). Social participation considered as meaningful in old age − the perceptions of senior housing residents in Finland. Ageing Int. 48, 1238–1258. (37359716). doi: 10.1007/s12126-023-09522-z, 37359716 PMC9989560

[ref86] Southerland J. L. Zheng S. Dodson K. Mauck E. Bacsu J.-D. Brown M. J. . (2024). Social isolation and loneliness prevention among rural older adults aging-in-place: a needs assessment. Front. Public Health 12, 1404869–1404869. doi: 10.3389/fpubh.2024.1404869 PMC11518758 39473600

[ref87] Sreepriya P. Balasubramanian R. (2020). Soil degradation in Kerala state and a case study on socio-economic impact due to flood in its Idukki district. J. Appl. Nat. Sci. 12, 159–164. doi: 10.31018/jans.vi.2265

[ref88] Srivastava S. Muhammad T. Paul R. Khan K. A. (2023). Multivariate decomposition of gender differentials in successful aging among older adults in India. BMC Geriatr. 23:59. doi: 10.1186/s12877-023-03753-0, 36721109 PMC9890860

[ref89] Stephens C. Breheny M. (2018). Healthy Ageing: A Capability Approach to Inclusive Policy and Practice. 1st Edn London: Routledge.

[ref90] Svendsen G. L. H. Svendsen G. T. (2000). Measuring social capital: the Danish co-operative dairy movement. Sociol. Rural. 40, 72–86. doi: 10.1111/1467-9523.00132

[ref91] Tanskanen A. O. Danielsbacka M. (2018). Intergenerational Family Relations. London: Routledge.

[ref92] Thomas P. A. Liu H. Umberson D. (2017). Family relationships and well-being. Innov. Aging 1:igx025. doi: 10.1093/geroni/igx025 PMC5954612 29795792

[ref93] Toyoshima A. Nakahara J. (2021). The effects of familial social support relationships on identity meaning in older adults: a longitudinal investigation. Front. Psychol. 12:650051. doi: 10.3389/fpsyg.2021.650051, 34113289 PMC8185044

[ref94] Ugargol A. P. Bailey A. (2020). Reciprocity between older adults and their care-givers in emigrant households of Kerala, India. Ageing Soc. 41, 1699–1725. doi: 10.1017/s0144686x19001685

[ref95] Vaughan G. Carson D. B. Preston R. Mude W. Holt A. (2022). A “toolkit” for rural aged care? Global insights from a scoping review. Front. Polit. Sci. 4:885636. doi: 10.3389/fpos.2022.885636

[ref96] Vogelsang E. M. (2016). Older adult social participation and its relationship with health: rural-urban differences. Health Place 42, 111–119. doi: 10.1016/j.healthplace.2016.09.010, 27755999 PMC5116414

[ref98] Wang L. Liu Y. Di X. Dai X. (2020). Family support, multidimensional health, and living satisfaction among the elderly: a case from Shaanxi Province, China. Int. J. Environ. Res. Public Health 17, 8434–8434. doi: 10.3390/ijerph17228434 PMC7696496 33202585

[ref99] Weng P.-Y. Li D. Liao M.-L. Chiang Y.-C. (2025). Aging in rural communities: engagement in indoor leisure activities and older adult health. PLoS One 20:e0334111. doi: 10.1371/journal.pone.0334111, 41086158 PMC12520377

[ref100] Wisnu A. (2015) Migration and Family Support: Case of Indonesian gay HAL (Le Centre Pour La Communication Scientifique Directe). Available online at: https://hal.archives-ouvertes.fr/tel-01231844

[ref101] Wong A. K. C. Liu T. Liu L. Bayuo J. Tao X. Wong F. K. Y. (2025). Healthcare and social needs of older adults in underserved urban communities: insights from community health workers. J. Urban Health 102, 1057–1068. doi: 10.1007/s11524-025-01011-9, 41015613 PMC12669452

[ref102] Yang Y. Huang Y. Huang J. Nie F. (2024). The role of social capital in the impact of multiple shocks on households’ coping strategies in underdeveloped rural areas. Sci. Rep. 14:14218. doi: 10.1038/s41598-024-65206-x, 38902363 PMC11190275

[ref103] Yasin Y. M. Al-Hamad A. Metersky K. Richard E. (2025). Uncovering key determinants of well-being among older Canadian retirees in New Brunswick: protocol for a mixed-methods study. PLoS One 20:e0334022. doi: 10.1371/journal.pone.0334022, 41071851 PMC12513576

[ref104] Yogendra N. D. (2024). Promoting sustainable livelihoods: evidence from the tribal regions of Marayoor, Kerala. Curr. Sci. 127, 153–153. doi: 10.18520/cs/v127/i2/153-159

[ref105] Zábó V. Csiszár A. Ungvári Z. Purebl G. (2023). Psychological resilience and competence: key promoters of successful aging and flourishing in late life. GeroScience 45, 3045–3058. (37418098). doi: 10.1007/s11357-023-00856-9, 37418098 PMC10643728

[ref106] Zeng Z. Xie G. He Y. Zhou S. (2025). How does intergenerational support from adult children shape the intrinsic capacity of older adults? Empirical evidence from the CHARLS. Aging Clin. Exp. Res. 37:268. doi: 10.1007/s40520-025-03175-5, 40886245 PMC12399442

[ref107] Zhang Y. Sun L. (2024). The health status, social support, and subjective well-being of older individuals: evidence from the Chinese general social survey. Front. Public Health 12:1312841. doi: 10.3389/fpubh.2024.1312841 PMC10850324 38333739

[ref108] Zhang C. Tang D. Wang Y. Jiang S. Liu X. (2022). Community support and promoting cognitive function for the elderly. Front. Psychol. 13:942474. doi: 10.3389/fpsyg.2022.942474, 36148108 PMC9488112

[ref109] Zhang J. Zhang Y. Wu Z. Fu X. (2024). Enhancing understanding of healthy aging based on time-varying dependencies among multidimensional health, life satisfaction, and health behaviors of older adults aged 60 years and over. BMC Public Health 24:192. doi: 10.1186/s12889-024-17752-2, 38229050 PMC10790531

[ref110] Zhao I. Y. Montayre J. Leung A. Y. M. Foster J. Kong A. Neville S. . (2022). Interventions addressing functional abilities of older people in rural and remote areas: a scoping review of available evidence based on WHO functional ability domains. BMC Geriatr. 22:827. doi: 10.1186/s12877-022-03460-2, 36307764 PMC9615260

[ref111] Zheng Z. Chen H. (2020). Age sequences of the elderly’ social network and its efficacies on well-being: An urban-rural comparison in China. BMC Geriatr. 20:372. doi: 10.1186/s12877-020-01773-8, 32993525 PMC7526405

[ref112] Zlotnick C. Groenou M. I. B. V. Orbán A. Corrigan T. Coimbrã S. Kirtava Z. . (2020). Lessons learned from 11 countries on programs promoting intergenerational solidarity. Fam. Relat. 70, 670–681. doi: 10.1111/fare.12482

